# Reduced miR-550a-3p leads to breast cancer initiation, growth, and metastasis by increasing levels of ERK1 and 2

**DOI:** 10.18632/oncotarget.10793

**Published:** 2016-07-23

**Authors:** Jar-Yi Ho, Ren-Jun Hsu, Chih-Hsi Wu, Guo-Shiou Liao, Hong-Wei Gao, Tong-Hong Wang, Cheng-Ping Yu

**Affiliations:** ^1^ Department of Pathology, and Graduate Institute of Pathology and Parasitology, Tri-Service General Hospital, National Defense Medical Center, Taipei, Taiwan; ^2^ Biobank Management Center of Tri-Service General Hospital, National Defense Medical Center, Taipei, Taiwan; ^3^ Department of Surgery, Tri-Service General Hospital, Taipei, Taiwan; ^4^ Tissue Bank, Chang Gung Memorial Hospital, Tao-Yuan, Taiwan

**Keywords:** miR-550a-3p, ERK1, ERK2, Ras/ERK signaling, breast cancer

## Abstract

Hyperactivation of the Ras/ERK pathway contributes to breast cancer initiation and progression, and recent evidence suggests aberrant signaling of miRNAs that regulate the Ras/ERK pathway play important roles during carcinogenesis and cancer progression. In this study, we demonstrate that miR-550a-3p expression is negatively correlated with levels of ERK1 and ERK2, two pivotal effectors in the Ras/ERK pathway. MiR-550a-3p gradually decreased during breast cancer initiation and progression and this reduction was a prognostic indicator of poorer overall survival (OS) and disease-free survival (DFS) among breast cancer patients. Our mechanistic studies demonstrated that miR-550a-3p exerts its tumor-suppressor role by directly repressing ERK1 and ERK2 protein expression, thereby suppressing the oncogenic ERK/RSK cascades, which reduced breast cancer cell viability, survival, migration, invasion, tumorigenesis, and metastasis. The inhibitory effects of miR-550a-3p were rescued by ectopic expression of ERK1 and/or ERK2. The novel connection between miR-550a-3p and ERK defines a new diagnostic and prognostic role for miR-550a-3p and highlights ERK inhibition as a candidate therapeutic target for breast cancers exhibiting hyperactivated Ras/ERK signaling.

## INTRODUCTION

Breast cancer is the most common female cancer and ranks among the leading causes of morbidity and mortality worldwide [1]; among Taiwanese women it occupies the top spot for morbidity and is fourth in mortality [2]. The RAS/extracellular signal regulated kinase (ERK) pathway is reported to enhance initiation and progression of several types of breast cancer and to promote cancer aggressiveness in a number of experimental models [3].

The Ras/ERK pathway (also known as the mitogen-activated protein kinase (MAPK)/ERK pathway or Ras-Raf-MEK-ERK cascade) is highly evolutionally conserved throughout the multicellular organisms [4] and plays an essential role in cancer initiation and progression [5, 6]. Ras/ERK hyperactivation is a common feature of a variety of tumor types featuring activating *KRAS*, *NRAS*, or *BRAF* gene mutations [7]; however, mutations in the pathway are detected in only ~3.2% of all breast lesions [8]. Rather, the Ras/ERK pathway is frequently activated as a consequence of alterations in upstream regulators or downstream effectors [5, 6]. Hyperactivation of the Ras/ERK pathway has been observed in approximately 50% of breast cancers [9, 10] and is significantly associated with advanced breast cancer progression and poorer prognosis [11-13]. MAPK1 (ERK2) and MAPK3 (ERK1) are pivotal effectors of the MAPK family which transduce mitogen-induced signals through the Ras/Raf/MEK/ERK cascade and trigger several important biological processes. Aberrant hyperactivation of ERK1 and 2 and their downstream targets/effectors is observed during cancer initiation, progression, and recurrence in a large subset of breast cancer [3, 5, 6]. Higher levels of ERKs1 and 2 and their active phosphorylated forms is significantly associated with increased risk of breast cancer incidence [14, 15] as well as poorer tumor differentiation, and larger tumor size [16], characteristic features of worse cancer progression or prognosis [11, 17, 18]. However, conflicting results have been reported [19, 20]. Despite the observation that *RAS* and *RAF* genes are rarely mutated, Ras/ERK signaling molecules are often overexpressed in breast cancer. For instance, ERK1 and ERK2 are overexpressed in 26%–45% of all molecular subtypes of breast cancer [21], which implies that the Ras/ERK pathway is more frequently activated by other mechanisms in breast cancer such as genomic or epigenetic variation of other pathway components [9, 11, 17].

MicroRNAs (miRNAs) are small non-coding RNAs with mature forms approximately 20–24 nucleotides in length, which may be involved in post-transcriptional regulation of oncogenes (oncomiR) or tumor suppressor genes (miRsupps) [22, 23]. miRNA dysregulation has been comprehensively documented in several breast cancer carcinogenic processes [24]. The miRNA dysregulation of Ras/ERK signaling molecules is largely associated with three Ras genes (KRAS, HRAS, and NRAS), such as occurs with the well-documented let-7 miRNA family [25]. Higher Ras levels should result in increased activation of downstream effectors. Dysregulation of several miRNAs involved in epigenetic activation of the Ras/ERK pathway have been investigated in different cancer types [26], but few studies have addressed miRNAs targeting Ras/ERK signaling molecules in breast cancer.

In this study, we sought to find miRNAs significantly downregulated in breast cancer by comparing miRNA expression in breast cancer and normal breast specimens using miRNA microarray. Our overall purpose was to determine the mechanism by which these miRNAs alter normal signaling cascades during breast cancer initiation and progression.

## RESULTS

### MiR-550a-3p is downregulated in breast cancer cell lines and tissues and is negatively correlated with ERK protein levels

**Figure 1:**
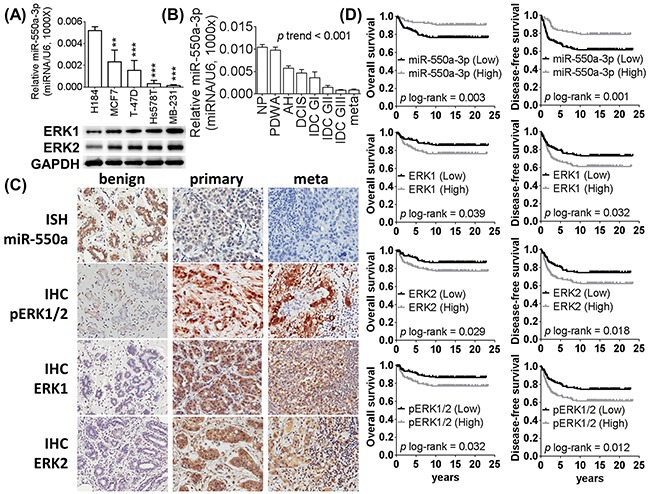
The expression of miR-550a-3p is downregulated in breast cancer and negatively corrected with ERK1 and ERK2 The relative expression levels of miR-550a-3p were detected by the stem-loop based miRNA real-time PCR (normalized to U6) in **A.** breast cell lines (H184B5F5/M10 was used as the baseline of comparison, *p < 0.05, **p < 0.01, ***p < 0.001, Student *t*-test). And ERK1 and ERK2 expression levels of each breast cancer cells were evaluated with western blot. **B.** The relative expression levels of miR-550a-3p were also detected by the stem-loop based miRNA real-time PCR in breast tissue specimens (twenty liquid-nitorgen storaged specimens of each group were analyzed, *p* trend test < 0.001, one-way ANOVA contrasts in a polynomial model). MiR-550a-3p was downregulated in breast cancer cell lines and tissues than normal controls. And lower miR-550a-3p was observed in poorer differentiated breast cancer cell lines or more aggressive breast cancer specimens. **C.** Representative expression profiles of miR-550a-3p, ERK1, ERK2, and p-ERK1/2 among formalin-fixed paraffin-embedded specimens of benign breast diseases, primary breast cancer and lymph node metastatic breast cancers. **D.** Kaplan-Meier curves of overall survival (OS) and disease-free survival (DFS) were analyzed with log-rank test. And higher miR-550a-3p (*p* = 0.003 for OS and *p* = 0.001 for DFS), lower ERK1 (*p* = 0.039 for OS and *p* = 0.032 for DFS), lower ERK2 (*p* = 0.029 for OS and *p* = 0.018 for DFS), and lower p-ERK1/2 (*p* = 0.032 for OS and *p* = 0.012 for DFS) carriers had better survival rate. Abbrev: NP: non-proliferative lesion, PDWA: proliferative disease without atypia, AH: atypical hyperplasia, DCIS: ductal carcinoma *in situ*, IDC GI: infiltrating ductal carcinoma grade I, IDC GII: infiltrating ductal carcinoma grade II, IDC GIII: infiltrating ductal carcinoma grade III, meta: lymph node metastatic breast cancer. ISH: *in situ* hybridization, IHC: immunohistochemistry.

To identify miRNAs involved in the breast cancer pathogenesis of our cohort of Taiwanese patients, we screened for the most downregulated tumor suppressor miRNA candidates in their breast cancer samples using miRNA microarray and validated with stem-loop real-time PCR. MiR-550a-3p was the most reduced of 105 identified significantly downregulated miRNAs (top 15 distinguishable miRNA are summarized in Supplementary Table S1). Similarly, miR-550a-3p was also significantly more highly expressed in the nontumorigenic human breast epithelial cell line H184B5F5/M10 than in breast cancer cell lines, and less miR-550a-3p was observed in poorly differentiated breast cancer cell lines (Figure 1A). Consistent results were observed in formalin-fixed, paraffin-embedded tissues; miR-550a-3p was expressed more highly in benign breast tissues but progressively lower in pre-neoplastic lesion (atypical hyperplasia, AH), more poorly differentiated primary tumors, and lymph node metastatic tumors (Figure 1B, *p* of trend test < 0.001).

To identify miRNAs targeting Ras/ERK signaling molecules, bioinformatics prediction was carried out for predicted partners for each member of the Ras/ERK signaling pathway. Interestingly, miR-550a-3p was predicted to target ERK1 and ERK2, which encouraged us to further evaluate the clinical relevance of miR-550a-3p and those two proteins during breast cancer initiation and progression. In Figure 1A, ERK1 and ERK2 are seen to display an inverse expression pattern with miR-550a-3p, in which higher ERK1 and ERK2 were observed in more poorly differentiated breast cancer cell lines and the lowest ERK1 and ERK2 were observed in H184B5F5/M10 cells. A case-control association study cohort comprising 300 breast cancer specimens and 300 benign breast disease specimens revealed miR-550a-3p to be significantly downregulated in primary breast cancer compared to benign breast diseases and nearly absent in metastatic tumors. In contrast, ERKs1 and 2, and their active phosphorylated forms (p-ERK1/2) were present in higher levels in metastatic breast cancer than primary breast cancer, and at very low levels in benign breast diseases (Figure 1C). Nuclear p-ERK1/2, reported to be the most deleteriously stained pattern, was dominantly observed in primary and metastatic breast cancer [16]. As shown in Supplementary Table S2, miR-550a-3p (*p* < 0.001) was significantly lower in breast cancer samples than benign breast disease samples; ERK1 (*p* = 0.036), ERK2 (*p* = 0.011) and p-ERK1/2 (*p* = 0.007), on the other hand, were significantly increased in the breast cancer samples relative to benign breast disease samples.

To evaluate their potential prognostic significance, survival analyses were performed for breast cancer patients with varying levels of miR-550a-3p and ERKs. Lower miR-550a-3p or higher ERK1, ERK2, and pERK1/2 were significantly associated with poorer overall survival (OS) and disease-free survival (DFS) for breast cancer patients (Figure 1D), suggesting that miR-550-3p reduction is a prognostic risk factor for both poorer OS and DFS in breast cancer.

### MiR-550a-3p reduces cell viability and increases apoptosis of breast cancer cells

**Figure 2:**
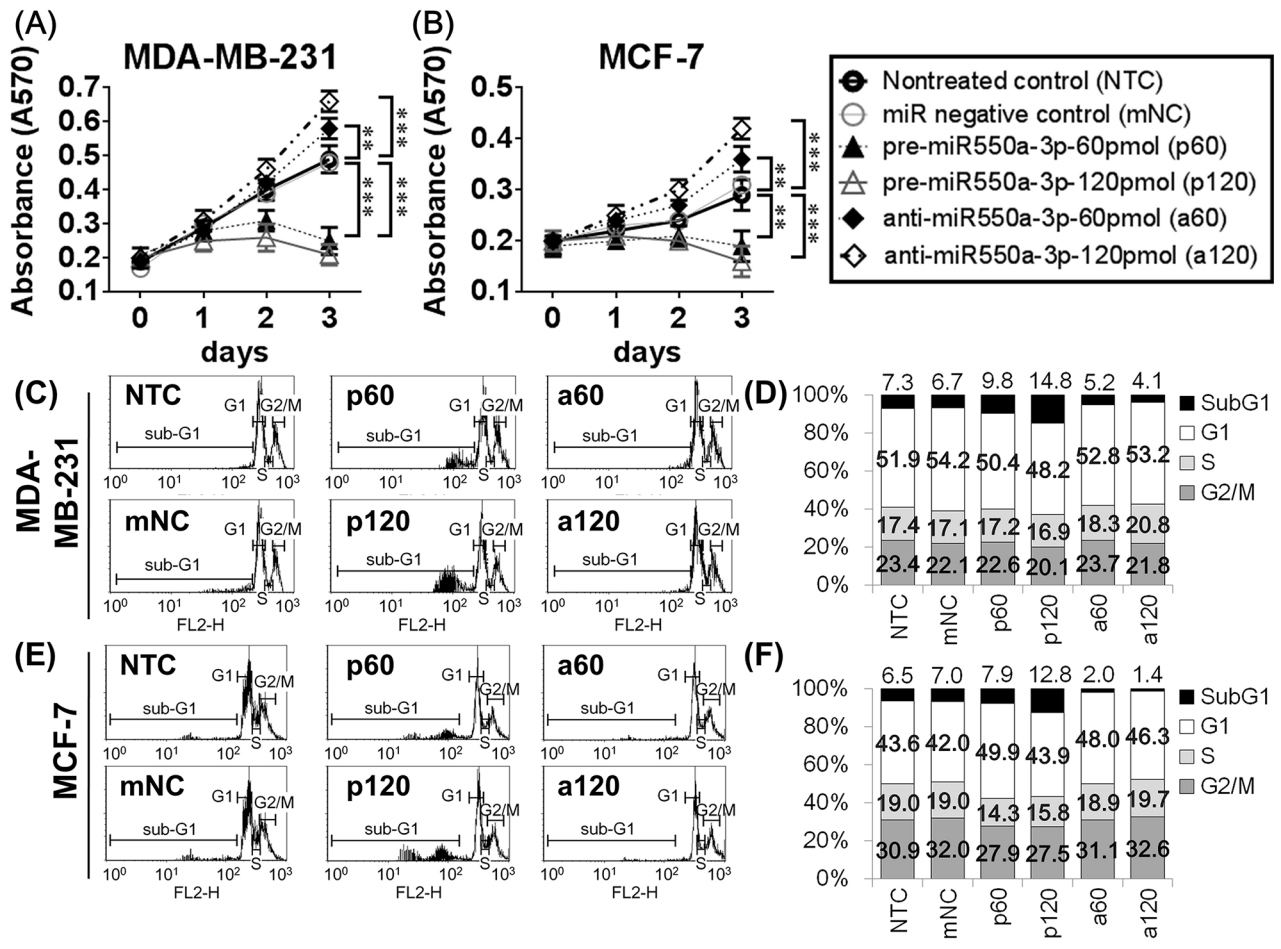
The effects of miR-550a-3p on cell viability and apoptosis were evaluated in MDA-MB-231 and MCF-7 cells **A.** MDA-MB-231 and **B.** MCF-7 cells were treated with six different conditions, non-treated control (NTC), 120 pmol miRNA negative controls (mNC), 60 pmol pre-miR-550a-3p (p60), 120 pmol pre-miR-550a-3p (p120), 60 pmol anti-miR-550a-3p (a60), and 120 pmol anti-miR-550a-3p (a120) for 72 h, and the cell viability was determined using MTT assay. It indicated that pre-miR-550a-3p inhibited cell viability in a dose-dependent manner, and otherwise anti-miR-550a-3p increased cell viability in a dose-dependent manner. **C, D.** MDA-MB-231 and **E, F.** MCF-7 cells were treated with the same six conditions, and sub-G1 area of PI-stained flowcytometry was considered as apoptotic cell population. It indicated that pre-miR-550a-3p increased sub-G1 area in a dose-dependent manner, and otherwise anti-miR-550a-3p reduced sub-G1 area in a dose-dependent manner.

The effects of miR-550a-3p on cell viability and apoptosis were evaluated in MDA-MB-231 and MCF-7 cells using MTT assay and propidium iodide (PI)-stained flow cytometry, respectively. Cells transfected with pre-miR-550a-3p were less viable than non-treated (NTC) or miRNA negative controls (mNC), and higher pre-miR-550a-3p dosage resulted in greater inhibition of cell viability. Conversely, transfection with anti-miR-550a-3p attenuated endogenous miR-550a-3p, resulting in higher cell viability than in the NTC or mNC groups, and higher anti-miR-550a-3p dosages resulted in greater increases in cell viability (Figure 2A, 2B). To confirm the effect of miR-550a-3p on the cell cycle, we quantified cyclin D1 and c-myc, two well-documented cell cycle markers, by western blot [27, 28]. Cells transfected with pre-miR-550a-3p showed a dose-dependent reduction in cyclin D1 and c-myc proteins, which was restored by transfection with anti-miR-550a-3p (Supplementary Figure S1A, S1B). Moreover, anti-miR-550a-3p had a smaller effect on cyclin D1 and c-myc in MDA-MB-231 cells, which have the lowest level of endogenous miR-550a-3p among the sampled breast cancer cell lines (Figure 1A). These data suggest that miR-550a-3p suppresses cell cycle entry and progression, resulting in reduced cell viability.

When evaluating apoptosis, transfection with pre-miR-550a-3p resulted in a larger sub-G1 population than NTC or mNC transfection, in a dose-dependent manner. Cells transfected with anti-miR-550a-3p had a smaller sub-G1 population than NTC or mNC groups in a dose-dependent manner (Figure 2C-2F). To confirm the effect of miR-550a-3p on apoptosis, cleaved caspase 3 and cleaved PARP were designated as apoptotic markers and Bcl-2 was designated as an anti-apoptotic marker [29, 30]. Cells transfected with pre-miR-550a-3p showed a dose-dependent increase in cleaved caspase 3 and cleaved PARP proteins, accompanied by a dose-dependent reduction in Bcl-2 (Supplementary Figure S1C, S1D). Conversely, cells transfected with anti-miR-550a-3p exhibited a dose-dependent reduction of cleaved caspase 3 and cleaved PARP and a dose-dependent increase in Bcl-2 expression. These data suggest that miR-550a-3p suppresses the expression of Bcl-2 and thereby induces apoptosis.

### MiR-550a-3p reduces breast cancer cell migration and invasion

**Figure 3:**
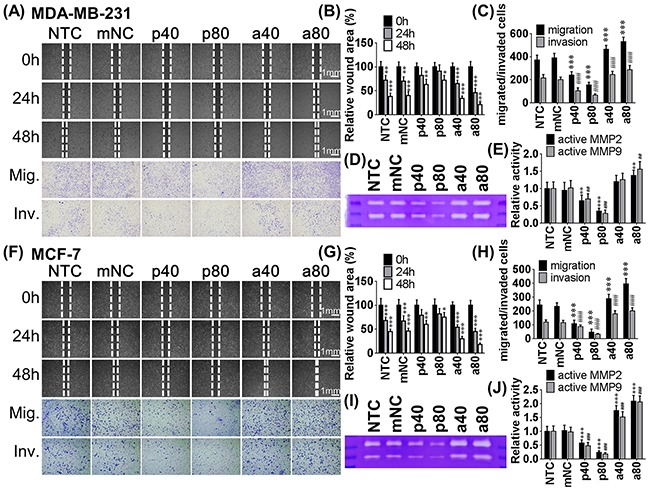
The effects of miR-550a-3p on cell motility were evaluated in MDA-MB-231 and MCF-7 cells Each cell was treated with six different conditions, non-treated control (NTC), 80 pmol miRNA negative controls (mNC), 40 pmol pre-miR-550a-3p (p40), 80 pmol pre-miR-550a-3p (p80), 40 pmol anti-miR-550a-3p (a40), and 80 pmol anti-miR-550a-3p (a80) for 72 h. Cell migratory abilities were analyzed with wound-healing and transwell assays, and cell invasive ability was analyzed with Matrigel-coated transwell assay. Lower concentration of pre-miR-550a-3p or anti-miR-550a-3p was used in these assays to reduce the interference of apoptosis. For wound healing assay, wound widths were recorded at 0, 24, and 48 h in **A.** MDA-MB-231 (line 1-3) and **F.** MCF-7 (line 1-3). Transwell assay and Matrigel-coated transwell assay were recorded at 48 h and 72 h, respectively, in (A) MDA-MB-231 (line 4-5) and (F) MCF-7 (line 4-5). Pre-miR-550a-3p reduced the cell migratory and invasive abilities but anti-miR-550a-3p promoted the cell migratory and invasive abilities in both cell lines. Wound-healing assay was determined with relative wound area of **B.** MDA-MB-231 and **G.** MCF-7, and transwell assays were determined the crystal violet stained migratated/invaded cell numbers of each group of **C.** MDA-MB-231 and **H.** MCF-7 (* *p* < 0.05, **p < 0.01, ***p < 0.001, Student *t*-tests were analyzed using the NTC group as baseline). Zymography also used to determine the effects of miR-550a-3p on MMP-2 and MMP-9 activities. In both **D, E.** MDA-MB-231 and **I, J.** MCF-7, pre-miR-550a-3p decreased active MMP-2 and active MMP-9 in a dose-dependent manner, and otherwise anti-miR-550a-3p increased active MMP-2 and active MMP-9 in a dose-dependent manner. Abbrev: Mig. migration, Inv. invasion.

To determine the effects of miR-550a-3p on cell motility, wound healing and transwell migration/invasion assays were employed; a lower concentration of pre-miR-550a-3p or anti-miR-550a-3p was used to reduce the interference of apoptosis. MDA-MB-231 and MCF-7 cells transfected with pre-miR-550a-3p exhibited a dose-dependent reduction of wound-healing ability relative to the NTC or mNC groups. Anti-miR-550a-3p conferred a dose-dependent increase in wound-healing ability compared to the NTC or mNC groups (Figure 3A upper, 3B, upper, 3G).

Similar results were observed in transwell migration assay; cells transfected with pre-miR-550a-3p exhibited a dose-dependent reduction of penetration ability through 8μm polyethylene membrane of transwell inserts compared to the NTC or mNC groups. Anti-miR-550a-3p, on the other hand, increased penetrating ability compared to the NTC or mNC groups in a dose dependent manner.

Matrigel-coated transwell assays were used to evaluate cell invasive ability. Cells transfected with pre-miR-550a-3p exhibited a dose-dependent reduced invasive ability on Matrigel-coated polyethylene transwell inserts compared to NTC or mNC groups. Anti-miR-550a-3p increased cells’ invasive ability in a dose-dependent manner compared to the NTC or mNC groups (Figure 3A lower, 3C, 3F lower, 3H).

ERK-induced MMP-2 and MMP-9 have been reported to degrade extracellular matrix, provoking cancer cell invasion into stroma [31, 32]; therefore, we used gelatin zymography to evaluate MMP-2 and MMP-9 activities at different miR-550a-5p concentrations. Cells transfected with pre-miR-550a-3p exhibited a dose-dependent reduction of MMP-2 and MMP-9 activities compared to NTC or mNC groups. Anti-miR-550a-3p increased MMP-2 and MMP-9 activities in a dose dependent manner compared to the NTC or mNC groups (Figure 3D, 3E, 3I, 3J). These data show that miR-550a-3p suppresses cell migration and invasion. Anti-miR-550a-3p was less effective in inhibiting cell motility in MDA-MB-231 cells due to their intrinsically lower miR-550a-3p.

### MiR-550a-3p reduces *in vitro* and *in vivo* tumorigenesis of breast cancer cells

**Figure 4:**
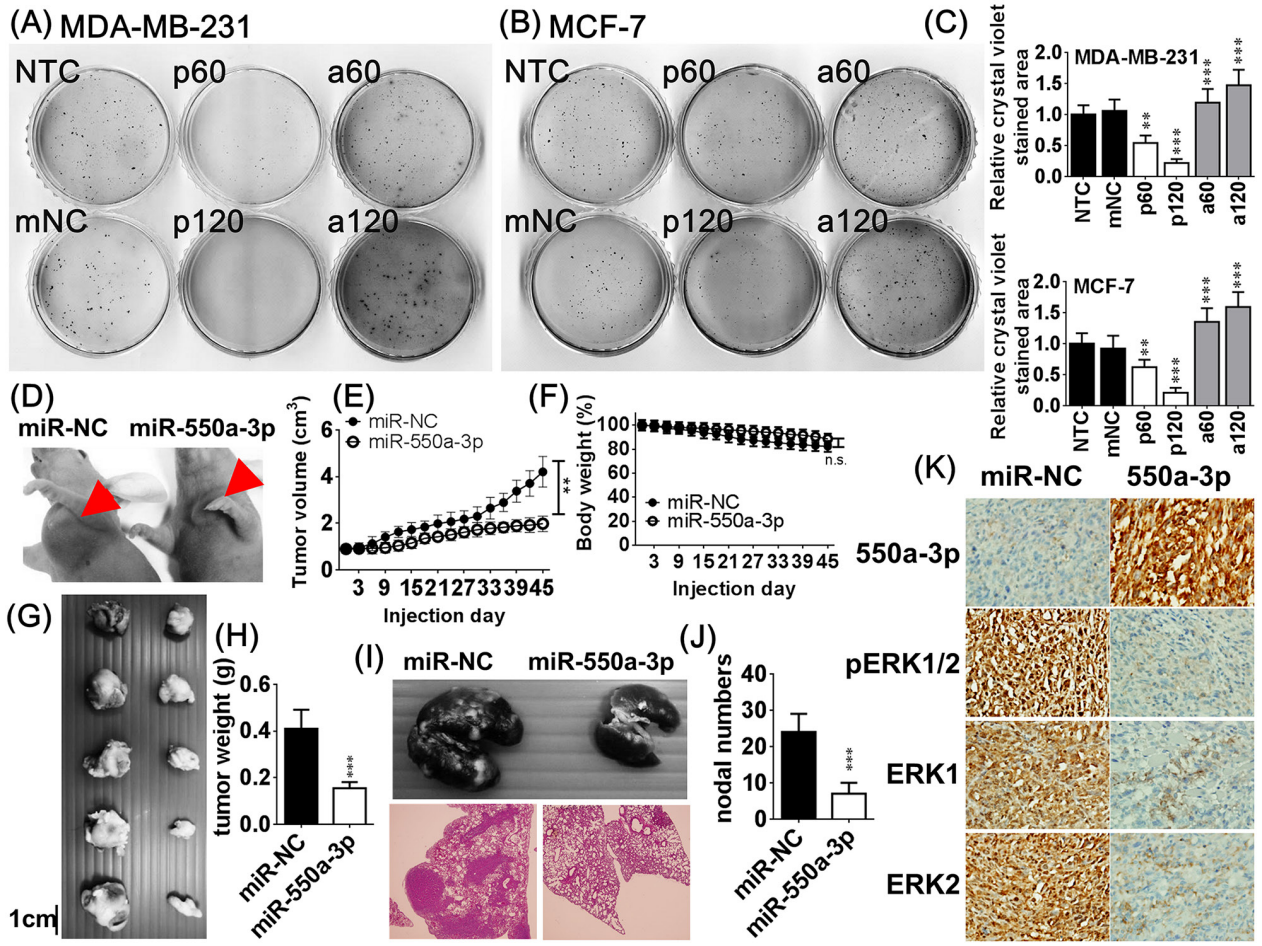
The effects of miR-550a-3p on *in vitro* and *in vivo* tumorigenesis After treated with the same six conditions of Figure 2 for 72 h, *in vitro* tumorigenesis was evaluated with softagar colony-forming assay in **A.** MDA-MB-231 and **B.** MCF-7. **C.** The bar-chart was used to show the colony numbers of each group and the NC group was used as the comparative group (*p < 0.05, **p < 0.01, ***p < 0.001, Student *t*-test). Pre-miR-550a-3p decreased colony numbers in a dose-dependent manner but anti-miR-550a-3p increased colony numbers in a dose-dependent manner. **D.** After MDA-MB-231 cells transfected with agomiR-550a-3p (5 μM) or agomiR-NC (5 μM) for 72 h, 1×10^7^ cells were injected subcutaneously and orthotopically into the axillary fossae of each female Balb/c nude mouse for 45 days (n = 5 for each group). **E.** AgomiR-550a-3p significantly reduced tumor volume but not modified **F.** body weight compared to the agomiR-NC transfected group. **G, H.** AgomiR-550a-3p also significantly reduced tumor weight compared to the agomiR-NC transfected group. Data are mean ± SD of quintuple experiments (**p < 0.01, ***p < 0.001, Student *t*-test). **I, J.** In metastasis assays, 2 × 10^7^ MDA-MB-231 cells transfected with agomiR-550a-3p (5 μM) or agomiR-NC (5 μM) were injected into the lateral tail veins of each mouse and allowed to colonize for 8 weeks (n = 5 for each group). AgomiR-550a-3p significantly reduced metastatic nodal numbers in lung compared to the agomiR-NC transfected group. Data are mean ± SD of quintuple experiments (**p < 0.01, ***p < 0.001, Student *t*-test). **K.** Representative results of *in situ* hybridization of miR-550a-3p and immunohistochemistry of ERK1, ERK2, and pERK1/2 in xenograft tumors.

Colony formation assay and a xenograft mouse model were used to examine the *in vitro* and *in vivo* effects of miR-550a-3p on tumorigenesis, respectively. Tumor cells transfected with pre-miR-550a-3p displayed a dose-dependent reduction in colony formation relative to NTC or mNC-transfected cells. Anti-miR-550a-3p conferred a dose-dependent increase in higher colony forming numbers relative to NTC or mNC groups (Figure 4A-4C).

MDA-MB-231 cells transfected with agomiR-550a-3p or agomiR-NC were injected subcutaneously into the axillary fossae of female Balb/c nude mice and allowed to grow for 45 days to mimic the orthotopic circumstance [33]. AgomiR-550a-3p significantly reduced tumor weight and volume (Figure 4D-4H) but not modified body weight compared to the agomiR-NC transfected group.

In metastasis assays, MDA-MB-231 cells transfected with agomiR-550a-3p or agomiR-NC were injected into the lateral tail veins of each mouse and allowed to colonize for 8 weeks (n = 5 for each group). AgomiR-550a-3p significantly reduced metastatic nodal numbers in lung. *In situ* hybridization of miR-550a-3p and immunohistochemistry of ERK1, ERK2, and pERK1/2 in xenograft tumors revealed a negatively correlated pattern between the expression of miR-550a-3p and the protein levels of ERK1, ERK2, and pERK1/2 (Figure 4I-4K). Together these data indicate that miR-550a-3p suppress *in vitro* and *in vivo* tumorigenesis and reduce metastatic ability of breast cancer cells.

### MiR-550a-3p directly targets ERK1 and ERK2

**Figure 5:**
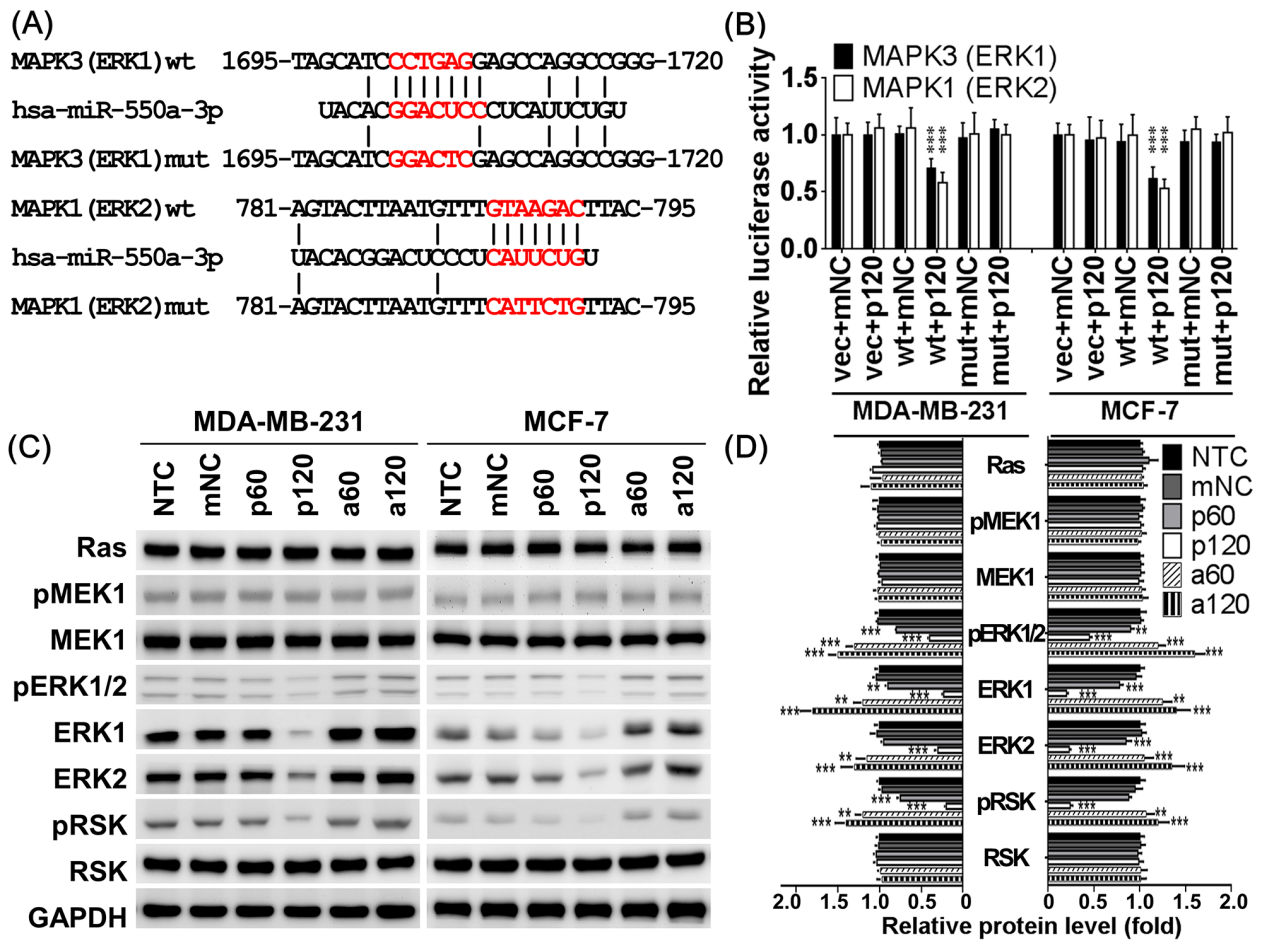
MiR-550a-3p downregulated ERK1 and ERK2 by directly targeting their 3’UTR **A.** The putative binding sequences for miR-550a-3p within the 3’UTR of *MAPK1* (ERK2) and *MAPK3* (ERK1) were aligned as indicated, and the mutated sites were designed upon the completely complementary sequences. **B.** After co-transfection with the pre-miR-550a-3p or miRNA negative controls (mNC), the relative luciferase activity of pMIR-Reporter luciferase reporters carring wild-type or mutant 3’UTR of *MAPK1* (ERK2) and *MAPK3* (ERK1) was determined in MDA-MB-231 and MCF-7 cells under normalization with the empty vector transfectants. β-Galactosidase control vector was also co-transfected as a transfection loading control. Pre-miR-550a-3p reduced luciferase activity in cells transfected with p-MIR-Reporter carrying wild-type ERK1 or ERK2 3’UTR, but there was no influence observed in cells transfected with vector alone orp-MIR-Reporter carryingmutantERK1 or ERK2 3’UTR. Data were shown as the mean ± SD. **C.** The protein levels of Ras/ERK signaling molecules were determined with western blot in MDA-MB-231 and MCF-7. Pre-miR-550a-3p decreased ERK1, ERK2, pERK1/2 and their downstream effect pRSK in a dose-dependent manner, but it conferred no effect on their upstream regulators, Ras, MEK1, and pMEK1. Otherwise, anti-miR-550a-3p slightly recovered the protein expression of ERK1 and ERK2 and therefore increased pERK1/2 and pRSK in a dose-dependent manner, but it conferred no effect on Ras, MEK1, and pMEK1. **D.** The bar-chart was used to show the relative protein levels which were normalized with GAPDH and the NTC group was used as the comparative baseline (*p < 0.05, **p < 0.01, ***p < 0.001, Student *t*-test). Abbrev: NTC: non-treated control, mNC: 120 pmol miRNA negative controls, p60: 60 pmol pre-miR-550a-3p, p120: 120 pmol pre-miR-550a-3p, a60: 60 pmol anti-miR-550a-3p, and p120: 120 pmol anti-miR-550a-3p.

The predicted miR-550a-3p targeting sites within the 3’UTR of ERK1 and ERK2 are shown in Figure 5A. Luciferase reporter assay showed that pre-miR-550a-3p reduced luciferase activity in cells transfected with p-MIR-Reporter carrying wild-type ERK1 or ERK2 3’UTR, but there was no influence observed in cells transfected with vector alone orp-MIR-Reporter carryingmutantERK1 or ERK2 3’UTR (Figure 5B).

To confirm the specificity of miR-550a-3p suppressive effects on ERK1 and ERK2, western blot was used to compare the major members of the Ras/MEK/ERK/90kDa ribosomal s6 kinase (RSK) cascade. As expected, miR-550a-3p transfected cells had significantly lower protein levels of ERK1, ERK2, and their active form pERK1/2. Consequently, miR-550a-3p also reduced the amount of phosphorylated RSK (pRSK), a dominant downstream effector of ERKs, without affecting its total protein (RSK). Moreover, pre-miR-550a-3p did not affect expression or activation of upstream regulators of the ERKs including Ras, MEK1, and pMEK1 (Figure 5C, 5D). These results suggest that miR-550a-3p specifically suppresses ERK1 and ERK2 by directly targeting their 3’UTRs and inhibits their downstream activation of RSK.

### Effects of ERK1 and ERK2 knockdown on cell viability, migration, and invasion

**Figure 6:**
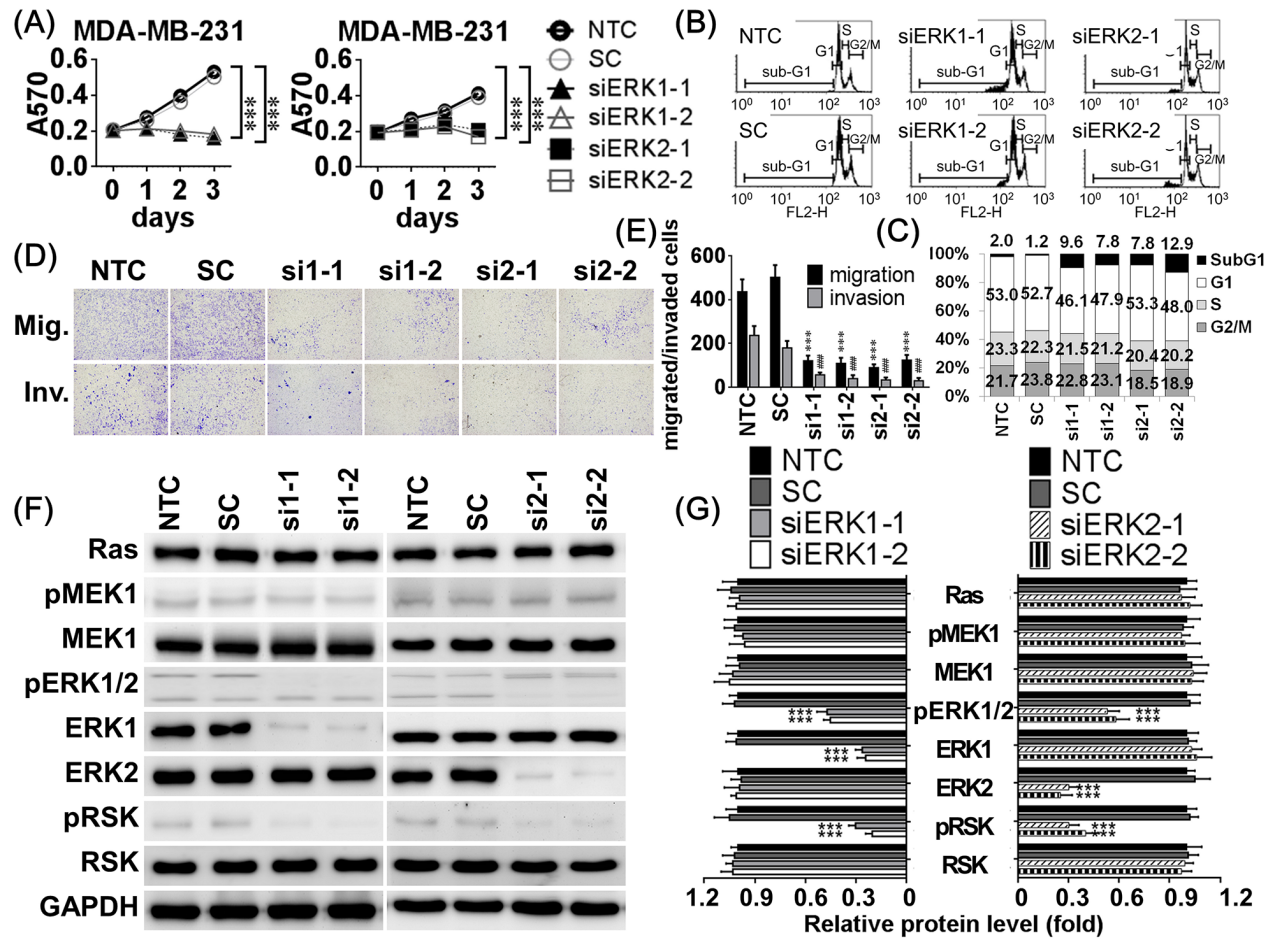
Knockdown ERK1 and ERK2 resulted in similar inhibitory effects of miR-550a-3p **A.** MDA-MB-231 cells were treated with six conditions, non-treated control (NTC), 120 pmol scrambled siRNA control (SC), 120 pmol siERK1-1 (si1-1), 120 pmol siERK1-2 (si1-2), 120 pmol siERK2-1 (si2-1), and 120 pmol siERK2-2 (si2-2) for 72 h, and the cell viability was determined using MTT assay. Those results indicated that knockdown of either ERK1 or ERK2 inhibited cell viability. **B, C.** Cells were treated with the same conditions for 72 h, and sub-G1 area of PI-stained flow cytometry was considered as apoptotic cell population. Those results indicated that knockdown of either ERK1 or ERK2 increased apoptosis. **D, E.** After treated with the same conditions for 72 h, cells were conducted to transwell or Matrigel-coated transwell assays to evaluate cell migratory and invasive abilities, respectively. Those results indicated that knockdown of either ERK1 or ERK2 inhibited cell migratory and invasive abilities compared to the NTC group (***p < 0.001 for migration, *p < 0.001 for invasion, Student *t*-test). **F.** The protein levels of Ras/ERK signaling molecules were determined with western blot. Those results indicated that knockdown of either ERK1 or ERK2 decreased the levels of ERK1, ERK2, pERK1/2, and pRSK without affecting their upstream regulators, Ras, MEK1, and pMEK1. **G.** The bar-chart was used to show the relative protein levels which were normalized with GAPDH and the NTC group was used as the comparative baseline (*p < 0.05, **p < 0.01, ***p < 0.001, Student *t*-test). The counterparts of MCF-7 were showed in Supplementary Figure S2.

To confirm ERK1 and ERK2 downregulation inhibited cell viability, migration, and invasion, siRNAs were used to knockdown ERK1 or ERK2. Each siRNA specifically inhibited its target gene (Figure 6F, Supplementary Figure S2F). Addition of either ERK1 or ERK2 siRNA reduced cell viability and induced higher sub-G1 apoptotic cell population in a dose-dependent manner (Figure 6A-6C, Supplementary Figure S2A-S2C). Cells transfected with either ERK1 or ERK2 siRNA exhibited less migratory and invasive abilities in transwell assays than NTC or SC groups (Figure 6D, 6E, Supplementary Figure S2D, S2E). Additionally, ERK1 or ERK2 siRNAs reduced downstream phosphorylation and activation of RSK. Neither ERK1 nor ERK2 siRNAs changed the expression or phosphorylation of their upstream regulators, Ras, and MEK1 (Figure 6F, 6G, Supplementary Figure S2F, S2G). These results show that direct knockdown of ERKs had suppressive effects similar to miR-550a-3p on cell viability, migration, and invasion.

### MiR-550a-3p inhibitory effects are attenuated by compensatory ERK expression

**Figure 7:**
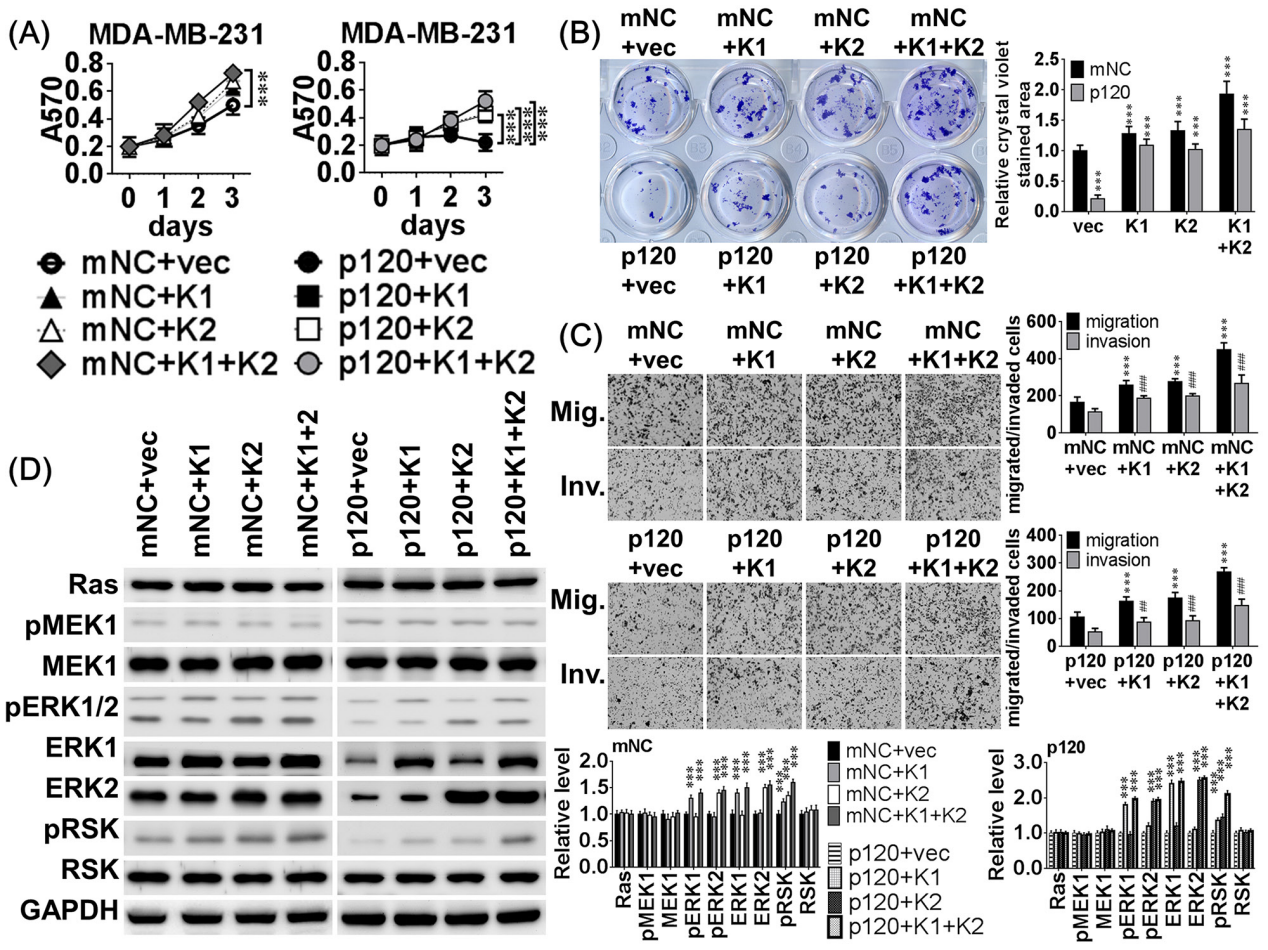
MiR-550a-3p inhibitory effects were attenuated by compensatory ERK expression **A.** MDA-MB-231 cells were transiently co-transfected with 8 different combinations of 120 pmol miRNA negative controls (mNC) or 120 pmol pre-miR-550a-3p combined with 2 μg of pcDNA3.1 empty vector, pcDNA3-ERK1, pcDNA3-ERK2, or both pcDNA-ERKs for 72 h. The inhibitory effects of miR-550a-3p on (A) cell viability, **B.** clonogenicity, **C.** migration and invasion were impaired in MDA-MB-231 cells co-transfected with pre-miR-550a-3p combined to ERK1 and/or ERK2. The growth curves and bar-charts were used to show the relative levels which were normalized to the mNC+vec groups (A&B: * *p* < 0.05, **p < 0.01, ***p < 0.001; and C: **p < 0.01, ***p < 0.001 for migration, *p <0.001 for invasion, Student *t*-test). **D.** The protein levels of the Ras/ERK signaling members were determined with western blot. And MDA-MB-231 cells co-transfected with pre-miR-550a-3p combined to ERK1 and/or ERK2 attenuated miR-550a-3p repressed Ras/ERK signaling effectors, pERK1/2 and pRSK. The bar-chart was used to show the relative protein levels which were normalized to GAPDH and the mNC+vec group was used as the comparative baseline (*p < 0.05, **p < 0.01, ***p < 0.001, Student *t*-test).

To examine whether compensatory expression of ERK1 or ERK2 attenuated the inhibitory effects of miR-550a-3p on breast cancer cell viability and progression, pcDNA3-ERK1, pcDNA3-ERK2 or empty vector were co-transfected into MDA-MB-231 (Figure 7) and MCF-7 (Supplementary Figure S3) cells with the pre-miR-550a-3p or mNC. Western blot analysis confirmed that the pre-miR-550a-3p markedly and specifically decreased both ERK1 and ERK2 expression, but pcDNA3-ERK1 or pcDNA3-ERK2 transfected cells retained an overexpressed pattern of ERK1 or ERK2 proteins (Figure 7D, Supplementary Figure S3D). The inhibitory effects of miR-550a-3 on cell viability, clonogenicity, migration, and invasion were impaired in MDA-MB-231 cells co-transfected with miR-550a-3p mimics and pcDNA3-ERK1 and/or pcDNA3-ERK2 (Figure 7A-7C, Supplementary Figure S3A-S3C). The co-transfection also significantly attenuated the miR-550a-3p induced activation of Ras/ERK signaling effectors (Figure 7D, Supplementary Figure S3D).

### Specific MEK/ERK pathway inhibitors cause inhibitory effects similar to miR-550a-3p

A parallel experiment was designed to compare the effect of PD98059 and U0126, specific inhibitors of the MEK/ERK pathway, to miR-550a-3p (Supplementary Figure S4). The repressive effects of PD98059 and U0126 on cell viability, apoptosis, migration and invasion were similar to those of pre-miR-550a-3p in both MDA-MB-231 and MCF-7 cells (Supplementary Figure S4A-S4C). PD98059 and U0126 also significantly reduced the activation of Ras/ERK signaling effectors (Supplementary Figure S4D). Taken together, these findings clearly reveal that ERK1 and ERK2 are the direct and functional targets of miR-550a-3p in the Ras/ERK signaling regulation.

## DISCUSSION

**Figure 8:**
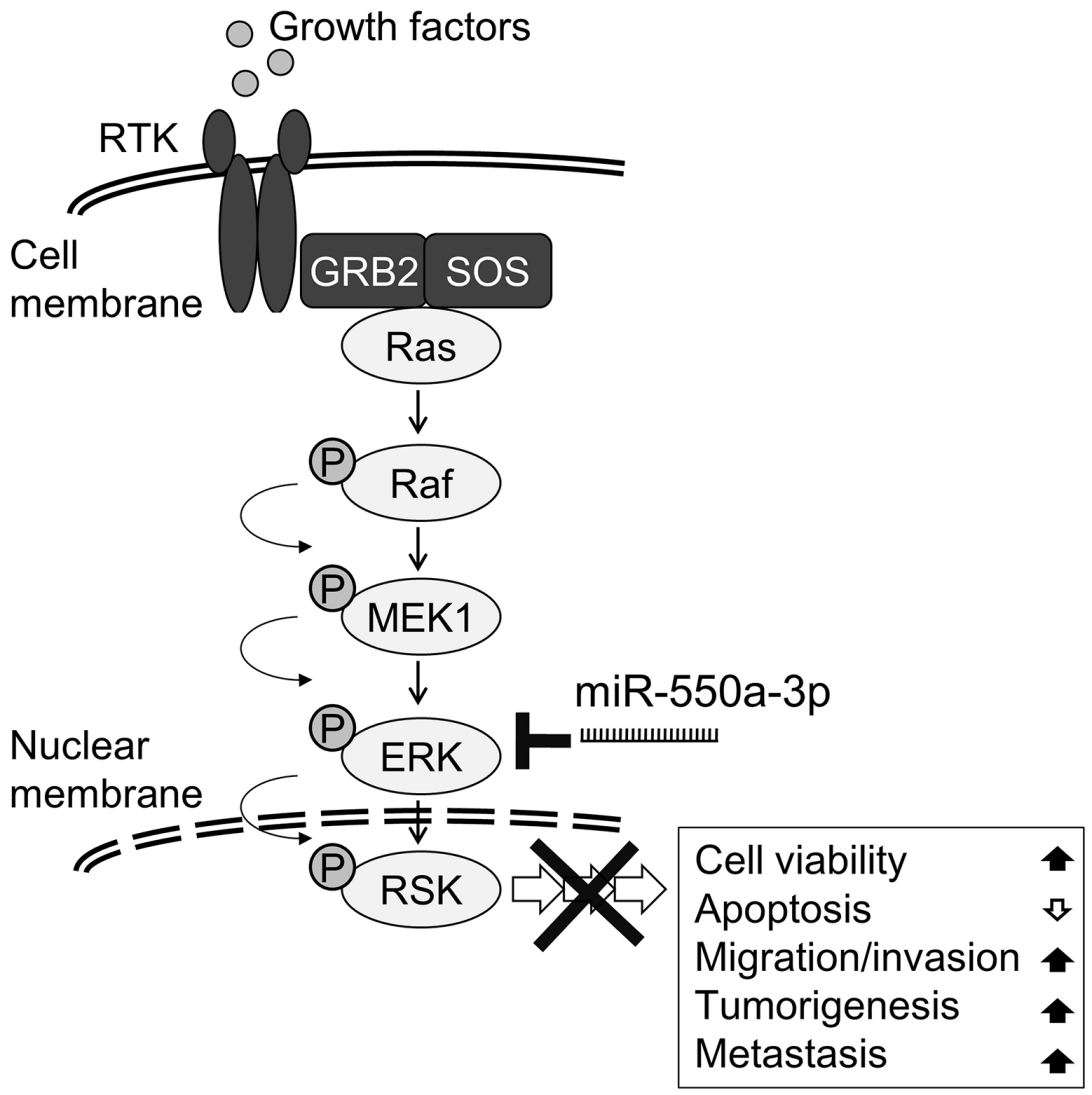
Schematic diagram of miR-550a-3p inhibits Ras/ERK signaling pathway by directly targeting ERK1 and ERK2 MiR-550a-3p exerts its tumor-suppressor role by directly targeting and repressing ERK1 and ERK2 and thereby suppresses the oncogenic ERK/RSK cascades, reducing cell viability, survival, migration, invasion, tumorigenesis, and metastasis in breast cancer cells.

In this study, we found the expression of the microRNA miR-550a-3p was negatively correlated with protein levels of ERK1 and ERK2, two pivotal effectors in the oncogenic Ras/ERK pathway, and ascribed significant diagnostic and prognostic values to the downregulation of miR-550a-3p during breast cancer initiation and progression. Our mechanistic studies demonstrate that miR-550a-3p exerts its tumor-suppressor role by directly targeting and repressing ERK1 and ERK2 and thereby suppresses the oncogenic ERK/RSK cascades [5, 34], reducing breast cancer cell viability, survival, migration, invasion, tumorigenesis, and metastasis (Figure 8). Rescue of the inhibitory effects of miR-550a-3p by ectopic ERK1 and/or ERK2 indicates that miR-550a-3p specifically inhibits both ERK1 and ERK2.

ERK1 and ERK2, which possess 85% protein sequence identity, are the most studied members of the MAPK family [35]. Both function as transducers of the well-characterized Ras/ERK pathway, which is triggered by growth factors and activating mutations of oncogenic kinases. Deregulation of the Ras/ERK pathway is observed in several cancers and results in changes in the expression of numerous genes involved in tumor cell differentiation, proliferation, survival, migration, and angiogenesis [5, 6]. In agreement with our findings, ERK1 and ERK2 overexpression and/or hyperactivation have been reported in a large subset of mammary tumors, and significantly predict higher risk of breast cancer incidence [14, 15]. Several survival analyses indicate that higher ERK1/2 expression or activity in primary breast tumors is prognostic for poorer overall survival or relapse-free survival of patients [11-13, 17, 18]. Higher nuclear p-ERK1/2 has also been associated with more aggressive behaviors of breast cancer such as higher pathological grade and larger tumor size [16], in agreement with our finding that nuclear p-ERK1/2 was the dominantly stained pattern in our primary tumor and metastatic breast cancer specimens. Moreover, the expression and activity of ERK1/2 also impact the patients’ therapeutic responses [36, 37]. Hence, most clinical interpretations highlight the relevance of ERK1/2 signaling to breast cancer; however, conflicting results have been reported [19, 20] and highlight the fact that the contradictory nature of ERK signaling in breast cancer remains unexplained.

Although RAS mutations are relatively infrequent in breast cancer [8], a constitutively activated Ras/ERK pathway is found in nearly half of breast tumors [10]. Similarly, active pERK1/2 was highly stained in ~38% of our recruited breast cancer specimens and positively correlated with higher expression of ERK1 (~30%) and ERK2 (~39%), in agreement with the trend seen in mRNA levels [21]. Moreover, miR-550a-3p repression of ERKs led to inhibition of cell proliferation and induction of apoptosis, which may result from inhibition of ERK downstream target genes, such as cyclin D1 [27], c-myc [28], and Bcl-2 [29].

Oncogenic RAS mutations occur in nearly 30% of all tumor types and mutations in other regulators or effectors are also common [38]. Those activating mutations partly explain why the Ras/ERK pathway is deregulated in approximately one-third of all human cancers [39]. Epigenetic alterations have also been reported to potentiate this activation during oncogenesis, especially those found to cause dysregulation of the miRNome [40]. *Let-7* miRNA pathway members have previously been reported to act as tumor suppressors by repressing the *RAS* oncogenes [25]. Various miRNAs have been reported to target members of the Ras/ERK pathway [26] and dysregulation of those miRNAs in cancer cells most likely contributes to tumorigenesis by causing aberrant activation of the Ras/ERK pathway.

To date, few studies have addressed miRNA targeting the Ras/ERK pathway in breast cancer. Let-7 family miRNAs have been well-documented to target *HRAS* to regulate self-renewal and tumorigenicity [41] and to sensitize *KRAS* mutant breast cancer to paclitaxel and gemcitabine [42]. In addition, miR-133a targeting of *EGFR*, miR-200c and miR-30c targeting of *KRAS*, miR-148b targeting of *NRAS*, miR-7 targeting of *RAF1*, miR-206 targeting of *RASA1* and miR-21 targeting of *SPRED1* have been reported in breast cancer cells [26]. However, this seems insufficient to explain why most Ras/ERK signaling mediators are overexpressed in breast cancer [21]. In this study, we explored miRNAs targeting Ras/ERK signaling mediators in breast cancer and found that miR-550a-3p directly targets and represses ERKs1 and 2. To our knowledge, only three miRNAs had been suggested to directly target ERKs: miR-483-5p targets *MAPK3 (ERK1)* in gliomas [43], miR-524-5p targets *MAPK1* (*ERK2)* in melanoma [44], and the miR-124/214 cluster targets both ERKs in squamous cell carcinoma; miR-214 targets *MAPK3 (ERK1)* whereas *MAPK1* (*ERK2)* is regulated by both miR-124 and miR-214 [45]. Here, we introduced a new connection between miR-550a-3p and ERK1/2 in breast cancer, the first study to address the regulatory role of miR-550a-3p.

MiR-550a-5p, a nearly antisense strand of miR-550a-3p, has a controversial role in carcinogenesis. It acts oncogenically by reducing two potential tumor suppressors, element binding protein 4 (*CPEB4*) in hepatocellular carcinoma [46] and ring finger 43 (*RNF43*) in colorectal cancer [47]; however, it also acts as a tumor-suppressor by directly targeting the 3’-UTR of the oncogene hepatocyte nuclear factor 1β (*HNF1B*) [48]. This first study of miR-550a-3p and for that matter the first study of any ERK-targeting miRNA in breast cancer suggests that miR-550a-3p may function in a different manner than its antisense partner, although both originate from the same pre-microRNA precursor. The regulatory interaction between miR-550a-3p and miR-550a-5p is still far-from understood and requires exploration in future studies.

In conclusion, miR-550a-3p plays a tumor-suppressor role in breast cancer initiation and progression by directly targeting and repressing ERK1 and ERK2 which inhibits activation of downstream ERK/RSK cascades and results in suppression of cancer cell viability, survival, migration, invasion, tumorigenesis and metastasis. Therefore, miR-550a-3p may be a useful diagnostic or prognostic marker in breast cancer. Ras/ERK pathway kinases are promising targets for identifying novel therapies [49]. Our findings indicate that miR-550a-3p represses activation of the Ras/ERK pathway in breast cancers and highlights that ERK inhibition may overcome acquired resistance to MEK inhibitors [50]. It also shows that ERK inhibitors or other agents targeting the ERK pathway members, such as agomiR-550a-3p, are potential treatments for cancers carrying hyperactivated Ras/ERK signaling.

## MATERIALS AND METHODS

### Subjects and tissue samples

The study population recruited subjects of 300 breast cancers and 320 benign breast diseases who were treated at Tri-Service General Hospital between 1992.06 and 2007.12. The recruited benign breast disease specimens comprised 237 nonproliferative lesions (NP, 124 fibroadenoma, 107 fibrocystic changes, 6 adenosis), 63 proliferative diseases without atypia (PDWA, 44 mild ductal hyperplasia and 19 sclerosing adenosis), and 20 atypical hyperplasias (AH, data only presented in Figure 1B). NP and PDWA reveal lower relative risk of breast cancer but AH are well-documented pre-neoplastic lesions [51]. And the recruited breast cancer specimens comprised 61 ductal carcinoma *in situ*, 27 infiltrating ductal carcinoma (IDC) grade I (GI), 110 IDC GII, 74 IDC GIII, 18 infiltrating lobular carcinoma (ILC), 7 colloid carcinoma, and 3 medullary carcinoma. In accordance of TSGH IRB regulations, at least eight-year postoperative follow-up was continued. The survival data were either obtained from the patient charts or the records of cancer registry council. These samples and information were obtained after informed consent according to institutional review board guidelines (TSGH-IRB-093-05-00004, 097-05-008, 098-05-204, 098-05-311, 099-05-273, and 100-05-236).

### MiRNA microarray and bioinformatical prediction

For miRNA microarray assay, liquid nitrogen stored spcimens comprised 5 breast cancer specimens (one infiltrating ductal carcinoma (IDC) grade I, two IDC grade II and two IDC grade III) and 5 normal breast specimens from benign breast diseases (two fibrocystic change and three fibroadenoma) were microdissected for RNA extraction. And miRNA microarray were performed with Agilent Human 8×15K miRNA Microarrays Rel18.0, which contains 43803 probes covering 1124 microRNAs, and the microarray data were analyzed using an Agilent Certified Service Provider Program (Welgene Biotech Co., Ltd., Taiwan). There were 105 significantly downregulated miRNAs (tumor/normal ratio > 1.5), and miR-550a-3p was the most reduced one. Using bioinformatic analysis with Microcosm Target, miRDB, TargetScan Human, and microRNA.org, we cross-compared the miRNA targeting Ras/ERK signaling genes and found that *MAPK1* (ERK2) is a predicted target of miR-550a-3p in several database. Besides, we also found that miR-550a-3p significantly downregulates *MAPK3* (ERK1) protein level but not affects the protein levels of Ras, Raf1, and MEK1. After an alignment-based screening for miR-550a-3p putative binding sites within *MAPK3* 3’UTR, we found there is a highly potential binding region as showed in Figure 6A.

### Cell lines

Human breast cancer cell lines MCF-7, T-47D, Hs578T, MDA-MB-231, and nontumorigenic human breast epithelial cell line H184B5F5/M10 were originally obtained from the Bioresource Collection and Research Center. Hs578T and MDA-MB-231 were maintained in Dulbecco's Modified Eagle medium containing 10% fetal bovine serum, 1μg/ml penicillin and 1μg/ml streptomycin (Invitrogen) at 37°C in a 5% CO_2_ atmosphere. MCF-7 and H184B5F5/M10 were maintained in MEM-α, and T-47D was maintained in Roswell Park Memorial Institute medium 1640 with the same supplements and culture condition. And two specific inhibitors of MEK/ERK pathway, PD98059 (50μM) and U0126 (20μM, Sigma-Aldrich Co), were used as parallel experiments for 72 h as the comparison of the effect of miR-550a-3p in both MDA-MB-231 and MCF-7 cells.

### RNA preparation and quantitative real-time PCR

Total RNAs of cells or specimens were isolated by TRIzol (Invitrogen) according to the manufacturer's instructions. For stem-loop based microRNA realtime PCR [52], five micrograms of isolated RNA was subjected to reverse transcription with SuperScript III (Invitrogen). SYBR Green based quantitative real-time PCR was processed by StepOne Real-Time PCR System (Applied Biosystems) using 2 × hot start PCR master mix (Applied Biosystems) in six repeats of each condition and U6 was used as an internal control. In Figure 1B, twenty of liquid-nitorgen storaged specimens of each group were analyzed.

### Western blot analysis

After washing with phosphate-buffered saline (PBS), treated cells were lyzed in 200 μl of radio-immunoprecipitation assay (RIPA) buffer (Millipore) containing protease inhibitor (Roche). 30 μg of protein from the cell lysate was loaded on SDS polyacrylamide gel followed by western blot analysis to detect the indicated protein levels (ERK1: Abcam ab9363, ERK2: Abcam ab32081, pERK1/2 (T202/Y204): Cell Signal Tech.#9422, cyclin D1: Cell Signaling Tech. #2926, c-myc: Santa Cruz Biotech.#sc-47694, cleaved PARP: Cell Signal Tech.#9422, cleaved caspase 3: Cell Signal Tech. #9664, Bcl-2: Epitomics #1017, Ras: Abcam ab52939, MEK1: Cell Signal Tech.#12671, pMEK1 (S218/222): Abcam ab32088, RSK: Cell Signal Tech. #9355, pRSK (T573): Cell Signal Tech. #9346, GAPDH: Epitomics #2251). The immuno-reactive bands were revealed by ECL system (Millipore) then developed and quantified on UVP BioSpectrum Imaging System, and each condition was done in three repeats.

### *In situ* hybridization

Deparaffinized specimens were peroxidase blocked with H_2_O_2_ and retrieved with proteonase XIV (0.125 ng/ml) for 1 hr at 37°C following fixation with 3.7% formaldehyde for 10 min at room temperature. Prepared specimens were transferred to a standard prehybridization solution (50% deionized formamide, 12.5% dextran sulphate, 0.3 M NaCl, 10 mM Tris-HCl pH 6.5, 5 mM EDTA, 0.1 M NaH_2_PO_4_, 1 mg/ml tRNA (Invitrogen), 1 × Denhardt's sloution (Sigma) in 1% DEPC treated H_2_O) for 1 h at 80°C to eliminate RNase activity. Pre-hybridizations were performed at 55°C for 1 h in a shaking hybridization oven. 100 pmol of probe (ATGTGCCTGAGGGAGTAAGACA-3’-biotin) was denatured at 99°C for 10 min following with hybridization at 55°C overnight. After triple washing with 0.2 × SSC at 55°C each for 1 h and rinsing with DEPC treated PBS for 10 min, each slide was incubated with HRP-conjugated Avidin (Dako) at room temperature for 1 h, incubated with DAB chromogen (Thermo Scientific) for 10 min and counterstained with hematoxylin.

### Immunohistochemistry

Immunohistochemistry was carried out in constructed tissue microarray containing 100 individual 2 mm-diameter samples per array. Each 4 μm section was blocked with 10% goat serum for 1 h and incubated with antibodies of indicated genes (ERK1: Abcam ab9363, ERK2: Abcam ab32081, pERK1/2 (T202/Y204): Cell Signal Tech.#9422) for 2 h at room temperature. After washing 3 times with TBST (10 mM Tris pH 7.4, 150 mM NaCl, 0.1% Tween-20) for 10 min, slides were processed by following the instructions of Super Sensitive Polymer HRP Detection System/DAB (Thermo Scientific) and counterstained with hematoxylin.

### Cell viability determined by MTT assay

Cells tranfected with indicated small RNA (pre-miR-550a-3p (Ambion #AM17100), anti-miR-550a-3p (Ambion #AM17000), miRNA negative control (mNC, Ambion #AM17110), ERK-1 siRNAs (Stealth siRNAs HSS108538 and HSS108539), ERK-2 siRNAs (Stealth siRNAs HSS108535 and HSS108536), Controls for Stealth RNAi siRNA (scrambled siRNA controls, SC, Stealth #12935-300, Thermo Fisher Scientific Inc., MA, USA)) for 72 h. After washing twice with PBS, 3000 cells of each treatment were seeded in 96 well plate overnight, and 3-(4,5-Dimethylthiazol-2-yl)-2, 5-diphenyl-tetrazolium bromide (MTT) assay was used to detect cell viability of those transfected cells or stably transfected clones.. In brief, 20 μl of 5 mg/ml MTT reagent were added to each well and incubated at 37°C for 3.5 h before reading absorbance at 570nm. A570 was recorded on 0 hr, 24 h, 48 h and 72 h, and each condition was done in six repeats.

### Flow cytometry

After trypsinizing and washing, 1x10^6^ cells were fixed with 100% ethanol for 10 min and incubated with 1 mg/ml propidium iodide (Sigma-Aldrich) for 10 min at room temperature. Cells were analyzed within 20 min post-staining on a BD FACSCalibur (BD Biosciences), and six repeats were performed for each condition.

### Migration/invasion assays

For wound-healing experiments, cells were plated in 6 cm dishes and cultured to >90% confluence. Cells were scraped with a p200 tip at time 0, the distances of migrating cells were measured from pictures (five fields) taken at the indicated time points using Image J software (NIH, USA). Each experiment was repeated six times. Transwell migration assay were assessed by 8μm inserts (BD Biosciences) with 1×10^4^ cells of each condition. And transwell invasion assays were evaluated with the same inserts coated with 1 mg/ml Matrigel (Invitrogen) with 2×10^4^ cells of each condition. The migration and invasion chambers were incubated in a humidified 5% CO_2_ incubator at 37°C for 48 hr. Cells were then fixed with methanol and wiped the inner surface of the upper chambers with cotton swabs to remove the un-migrated cells. After washing, the chambers were stained with crystal violet and the transwell membranes were torn and kept in slides. The crystal violet stained area were analyzed using Image J software and five random fields were counted at 100 × magnification, and each condition was done in six repeats.

### Construction of plasmids and luciferase reporter assay

R777-E125 Hs.MAPK1 was a gift from Dominic Esposito (Addgene plasmid # 70409) and GFP-ERK1 (*MAPK3*) was a gift from Rony Seger (Addgene plasmid # 14747). *MAPK1* (ERK2) and *MAPK3* (ERK1) open-reading frames were subclone into pcDNA3.1/His A vector (Invitrogen) within the *Not*I and *Xba*I restriction sites. All constructs were verified by auto-sequencing.

Potential miR-550a-3p binding sites in 3’UTR of ERK1 and ERK2 were aligned in Figure 6A. Human 3’-UTRs of miR-550a-3p target mRNAs were amplified by PCR from human genomic DNA using the primer pairs as followed: *MAPK1* (ERK2), F: tcctccactagTTCCCCAGAGCAGGAGCTT and R: tcctccaagcttGGGACATCCCCAGAAACC, to produce a 400bp insert. And *MAPK3* (ERK1), F: tcctccactagtTGCCTGCCCCTCTCC and R: tcctccaagcttTGGCAGGGGCGCCGGG, to produce a 544 bp insert. And mutant miR-550a-3p binding sites were designed as showed in Figure 6A. PCR fragments were restricted and ligated to a compatible *Hin*dIII and *Spe*I-linearized pMIR-Reporter vector (Invitrogen). A total of 2×10^5^ MDA-MB-231 or MCF-7 cells were seeded in 6 cm dishes 16 hr before transfection. 120 pmol of pre-miR-550a-3p or miRNA negative controls were co-transfected with 1 μg of pMIR-Reporter empty vector, pMIR-Reproter carried each wild-type ERK-3’UTR, and pMIR-Reproter carried each mutant ERK-3’UTR using Lipofectamine 2000 (Invitrogen). And 500 ng of β-gal Control vector were also co-transfected as an internal control. Luciferase assays were performed with Dual-Light Luciferase & β-Galactosidase Reporter Gene Assay System (Invitrogen), and each condition was done in six repeats.

### *In vivo* tumor xenograft and metastasis assays

Female athymic Balb/c nude mice (6-weeks old) were purchased from the National Laboratory Animal Center and acclimated for 1 week under conditions approved by the The Laboratory Animal Center of National Defense Medical Center. The biological effects of miR-550a-3p *in vivo* were determine as described [33], in brief, 1×10^7^ MDA-MB-231 cells transfected with agomiR-550a-3p (5 μM) or agomiR-NC (5 μM, Ribobio Co), respectively, were suspended in 100 μl PBS for each mouse and were injected subcutaneously and orthotopically into the axillary fossae of the female nude mice (5 mice per group). Tumor diameters were measured every 3 days. Mice were sacrificed at 45 days after injection, and tumors were weighted after necropsy. Tumor volume was calculated as follows: length × width^2^ × 1/2. For *in vivo* pulmonary metastasis assays, 2 × 10^7^ MDA-MB-231 cells transfected with agomir-550a-3p (5 μM) or agomiR-NC (5 μM), respectively. The cells were injected into the lateral tail veins of each nude mouse (5 mice per group). Mice were sacrificed at 8 weeks after injection, and lungs were fixed with phosphate-buffered neutral formalin before paraffin embedding. 4 μm sections were stained with hematoxylin and eosin or used for immunohistochemistry.

### Data analysis

Real-time PCR original data were quantifed with StepOne Sofeware Ver.2.2.2 and western blot data were quantified with Image J. These data were recorded as continuous variants and analyzed with Student's *t* test or linear polynomial ANOVA with LSD posthoc examination. Data of in situ hybridization or immunohistochemistry were quantified with Aperio ImageScope and Spectrum Ver. 10.0. Survival assays were evaluated Kaplan-Meier curve with log-rank test in the breast cancer patients. All the statistical analyses were performed using SPSS 16.0 and Excel 2010. All statistical tests and *p* values were two-sided and the level of significance was set at < 0.05 (*), < 0.01 (**), or < 0.001 (***).

## SUPPLEMENTARY MATERIALS FIGURES AND TABLES


