# Supplementary material for: Reduced miR-550a-3p leads to breast cancer initiation, growth, and metastasis by increasing levels of ERK1 and 2
Source: Oncotarget. 2016 Jul 23;7(33):53853–68. doi: 10.18632/oncotarget.10793 (PMC5288226; doi:10.18632/oncotarget.10793)
Supplement: Supplementary file 1 [file oncotarget-07-53853-s001.pdf]

Reduced miR-550a-3p leads to breast cancer initiation, growth, and metastasis by increasing levels of ERK1 and 2

SUPPLEMENTARY FIGURES AND TABLES

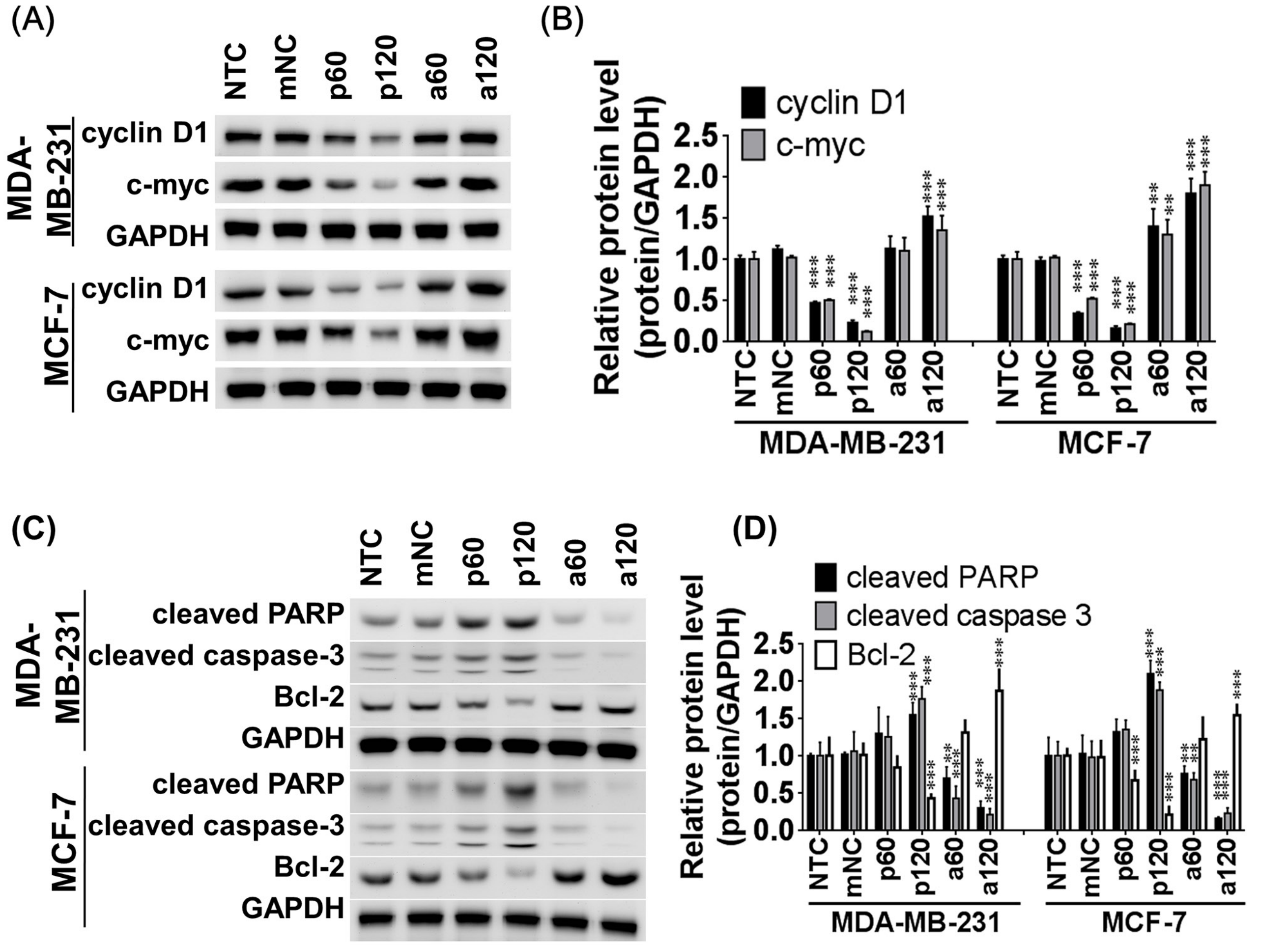

Supplementary Figure S1: A. Cyclin D1 and c-myc were designated as cell-cycle markers. Cells transfected with pre-miR-550a-3p showed a dose-dependent reduction in cyclin D1 and c-myc proteins, which was restored by transfection with anti-miR-550a-3p. **B.** The bar-chart was used to show the relative protein levels which were normalized with GAPDH and the NTC group was used as the comparative baseline (*$p < 0.05$, **$p < 0.01$, ***$p < 0.001$, Student *t*-test). **C.** Cleaved caspase 3 and cleaved PARP were designated as apoptotic markers and Bcl-2 was designated as an anti-apoptotic marker. Cells transfected with pre-miR-550a-3p showed a dose-dependent increase in cleaved caspase 3 and cleaved PARP proteins, accompanied by a dose-dependent reduction in Bcl-2. And cells transfected with anti-miR-550a-3p exhibited a dose-dependent reduction of cleaved caspase 3 and cleaved PARP and a dose-dependent increase in Bcl-2 expression. **D.** The bar-chart was used to show the relative protein levels which were normalized with GAPDH and the NTC group was used as the comparative baseline (*$p < 0.05$, **$p < 0.01$, ***$p < 0.001$, Student *t*-test).

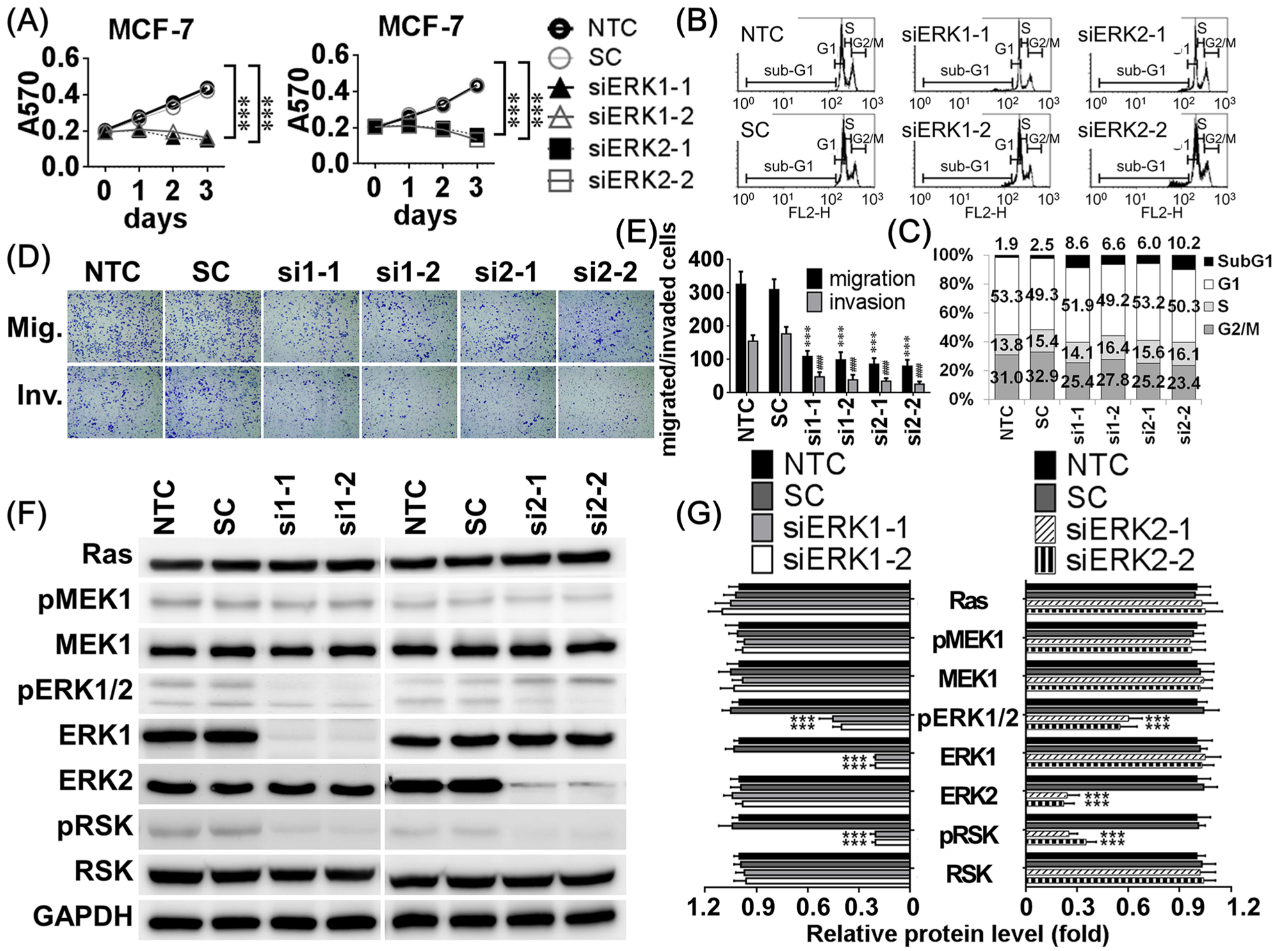

Supplementary Figure S2: Knockdown ERK1 and ERK2 resulted in similar inhibitory effects of miR-550a-3p in MCF-7 cells. A. MCF-7 cells were treated with the same six conditions of Figure 7 for 72 h, and the cell viability was determined using MTT assay. Those results indicated that knockdown of either ERK1 or ERK2 inhibited cell viability. **B, C.** Cells were treated with the same conditions for 72 h, and sub-G1 area of PI-stained flowcytometry was considered as apoptotic cell population. Those results indicated that knockdown of either ERK1 or ERK2 increased apoptosis. **D, E.** After treated with the same conditions for 72 h, cells were conducted to transwell or Matrigel-coated transwell assays to evaluate cell migratory and invasive abilities, respectively. Those results indicated that knockdown of either ERK1 or ERK2 inhibited cell migratory and invasive abilities compared to the NTC group (***p < 0.001 for migration, ###p <0.001 for invasion, Student t-test). **F.** The protein levels of Ras/ERK signaling molecules were determined with western blot with the same conditions. Those results indicated that knockdown of either ERK1 or ERK2 decreased the levels of ERK1, ERK2, pERK1/2 and pRSK without affecting their upstream regulators, Ras, MEK1, and pMEK1. **G.** The bar-chart was used to show the relative protein levels which were normalized with GAPDH and the NTC group was used as the comparative baseline (*p < 0.05, **p < 0.01, ***p < 0.001, Student t-test).

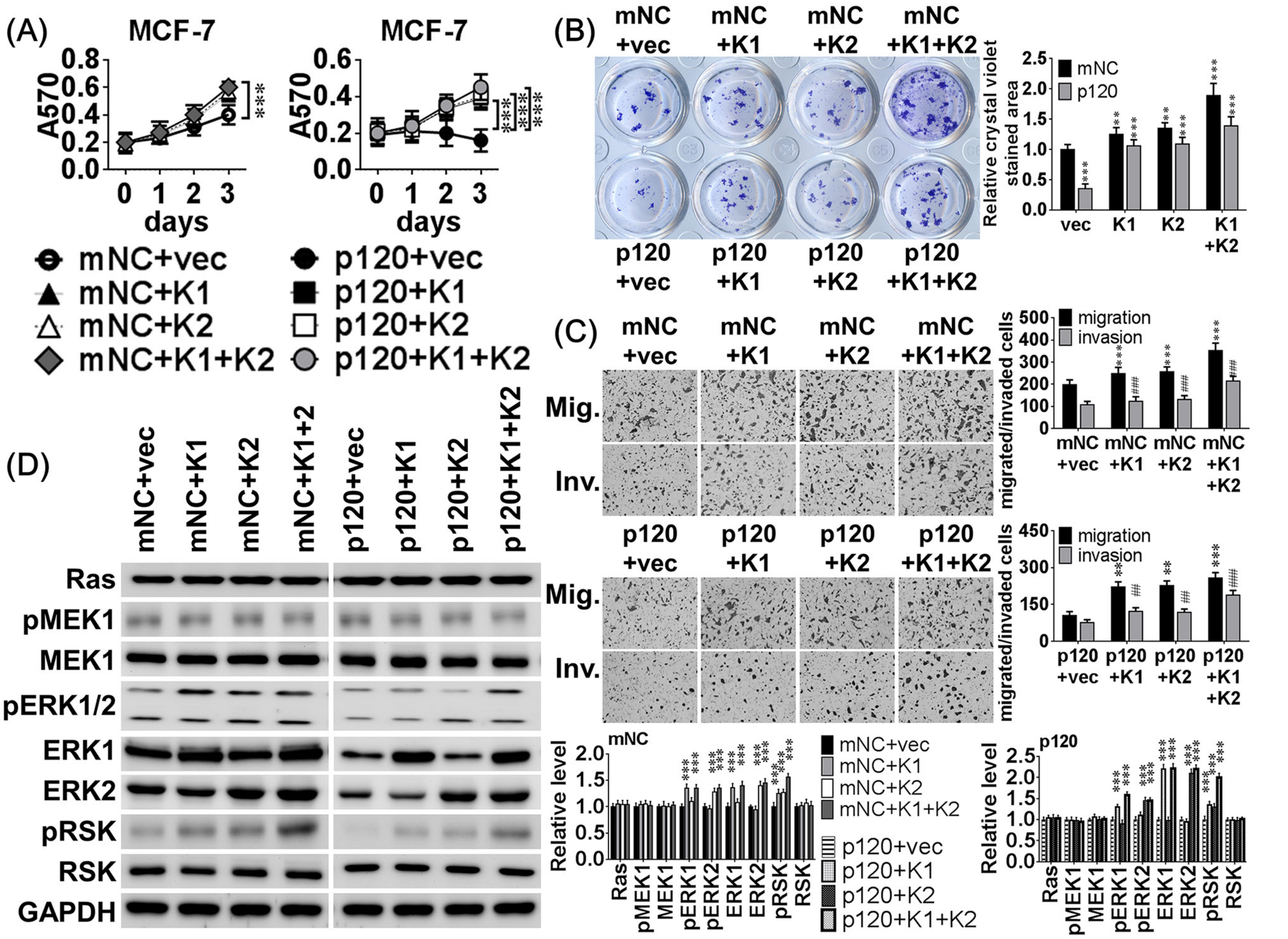

Supplementary Figure S3: MiR-550a-3p inhibitory effects were attenuated by compensatory ERK expression. A. MCF-7 cells were transiently co-transfected with 8 different combinations of 120 pmol miRNA negative control (mNC) or 120 pmol pre-miR-550a-3p combined with 2 µg of pcDNA3.1 empty vector, pcDNA3-ERK1, pcDNA3-ERK2, or both pcDNA-ERKs for 72 h. The inhibitory effects of miR-550a-3p on (A) cell viability, **B.** clonogenicity, **C.** migration and invasion were impaired in MCF-7 cells co-transfected with pre-miR-550a-3p combined to ERK1 and/or ERK2. The growth curves and bar-charts were used to show the relative levels which were normalized to the mNC+vec groups (A&B: * $p < 0.05$, ** $p < 0.01$, *** $p < 0.001$; and C: ** $p < 0.01$, *** $p < 0.001$ for migration, ### p <0.001 for invasion, Student *t*-test). **D.** The protein levels of the Ras/ERK signaling members were determined with western blot. And MCF-7 cells co-transfected with pre-miR-550a-3p combined to ERK1 and/or ERK2 attenuated miR-550a-3p repressed Ras/ERK signaling effectors, pERK1/2 and pRSK. The bar-chart was used to show the relative protein levels which were normalized to GAPDH and the mNC+vec group was used as the comparative baseline (* $p < 0.05$, ** $p < 0.01$, *** $p < 0.001$, Student *t*-test).

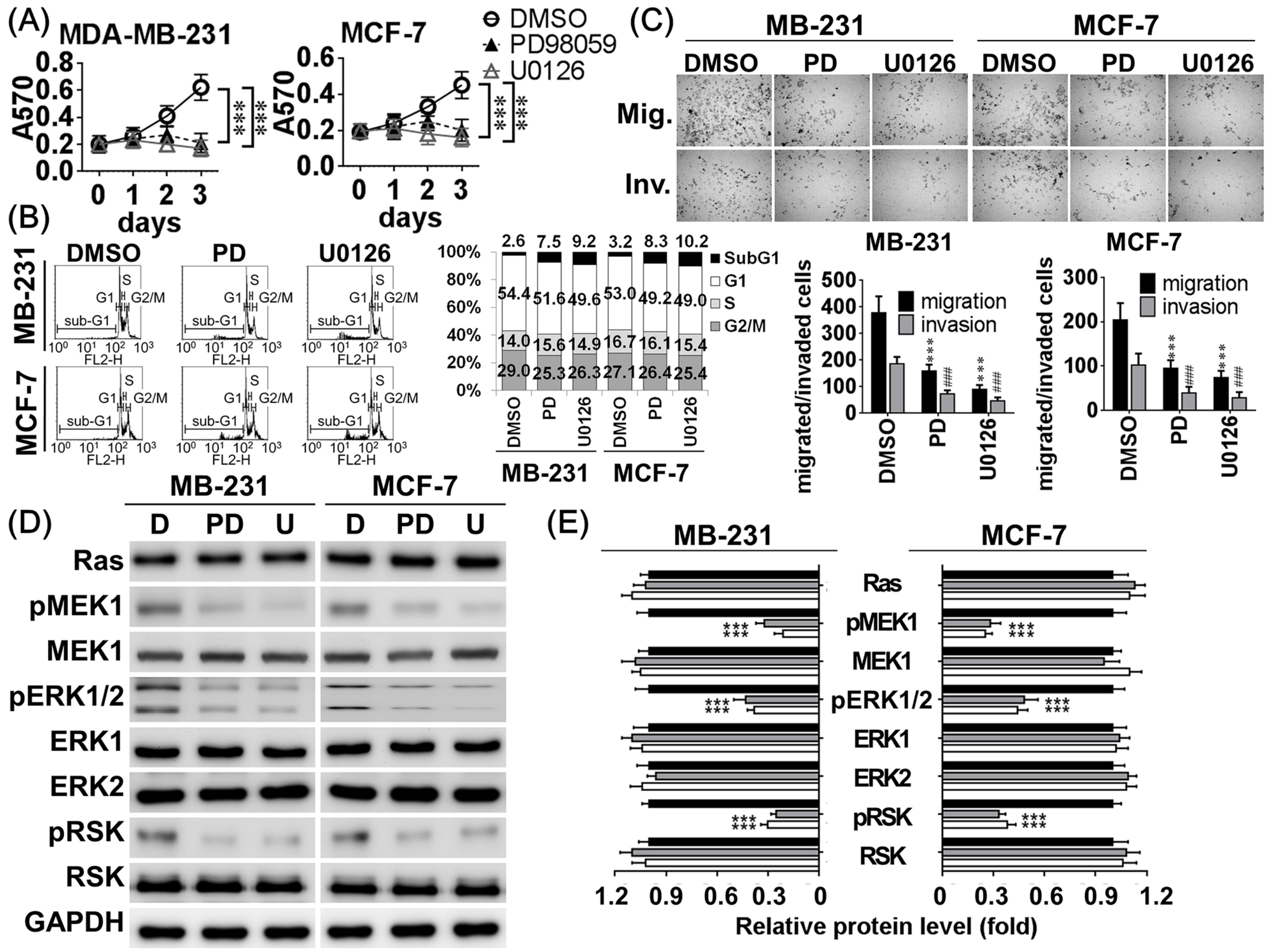

Supplementary Figure S4: Specific MEK/ERK pathway inhibitors cause inhibitory effects similar to miR-550a-3p. A parallel experiment was designed to compare the effect of PD98059 and U0126, specific inhibitors of the MEK/ERK pathway, to miR-550a-3p The repressive effects of PD98059 (50 µM) and U0126 (20 µM) on cell viability, apoptosis, migration and invasion were similar to those of pre-miR-550a-3p in both MDA-MB-231 and MCF-7 cells. PD98059 and U0126 also significantly reduced the activation of Ras/ERK signaling effectors, p-MEK, p-ERK1/2, and p-RSK. The bar-chart was used to show the relative protein levels which were normalized to GAPDH and the DMSO solvent group was used as the comparative baseline (*$p < 0.05$, **$p < 0.01$, ***$p < 0.001$, Student t-test).

Supplementary Table S1: Top 10 distinct miRNAs between breast carcinoma and normal breast tissue (microarray data)

	Breast cancer	Normal breast	Cancer/normal	*p*
Tumor suppressive miRNA				
hsa-miR-550a-3p	0.08	16.44	0.0049	<0.001
hsa-miR-15b	0.07	12.59	0.0056	<0.001
hsa-miR-132	0.09	10.48	0.0086	<0.001
hsa-miR-212	0.08	8.44	0.0095	<0.001
hsa-miR-153	0.08	8.13	0.0098	<0.001
hsa-miR-200c	0.09	7.21	0.0125	<0.001
hsa-miR-924	0.11	7.61	0.0145	<0.001
hsa-miR-105	0.12	6.28	0.0191	<0.001
hsa-miR-190b	0.11	5.30	0.0208	<0.001
hsa-miR-552	0.11	5.07	0.0217	<0.001
hsa-miR-145	0.28	5.47	0.0512	<0.001
hsa-miR-7-1	0.37	4.80	0.0771	<0.001
hsa-miR-143	0.44	5.12	0.0859	<0.001
hsa-miR-1301	0.95	4.43	0.2144	<0.001
hsa-miR-1244	1.10	4.60	0.2391	<0.001
Onco-miR				
hsa-miR-382-5p	254.91	0.11	2317.36	<0.001
hsa-miR-410	100.57	0.12	838.08	<0.001
hsa-miR-1254	64.47	0.15	429.80	<0.001
hsa-miR-381	137.43	0.71	192.48	<0.001
hsa-miR-154	95.42	0.58	165.95	<0.001
hsa-miR-136-3p	75.54	1.48	50.90	<0.001
hsa-miR-654-3p	163.12	11.44	14.26	<0.001
hsa-miR-92a-1	512.79	39.67	12.93	<0.001
hsa-miR-21-3p	68.20	5.35	12.75	<0.001
hsa-miR-204	66.05	5.57	11.86	<0.001
hsa-miR-155	81.66	7.43	10.99	<0.001
hsa-miR-21-5p	31540.60	3171.58	9.94	<0.001
hsa-miR-125b-2	138.81	16.32	8.51	<0.001
hsa-miR-542-3p	97.29	12.89	7.55	<0.001
hsa-miR-214	74.95	12.13	6.18	<0.001

Supplementary Table S2: miR-550a-3p and ERK expression profiles among benign breast diseases (BBD) and breast cancers (BC)

	BBD (n=300)	BC (n=300)	*p*[a]
**miR-550a-3p**			
**-low**	148	191	<0.001
**-high**	152	109	
**ERK1**			
**-low**	196	171	0.036
**-high**	104	129	
**ERK2**			
**-low**	172	141	0.011
**-high**	128	159	
**pERK1/2**			
**-low**	179	146	0.007
**-high**	121	154	

[a] On the basis of the chi-square-test.
BBD comprised 237 nonproliferative lesions (124 fibroadenoma, 107 fibrocystic changes, 6 adenosis) and 63 proliferative diseases without atypia (44 mild ductal hyperplasia and 19 sclerosing adenosis).
BC comprised 61 ductal carcinoma *in situ*, 27 infiltrating ductal carcinoma (IDC) grade I (GI), 110 IDC GII, 74 IDC GIII, 18 infiltrating lobular carcinoma (ILC), 7 colloid carcinoma, and 3 medullary carcinoma.
Expression profiles of miR-550a-3p were evaluated using *in situ* hybridization and expression profiles of ERK1, ERK2, and pERK1/2 were evaluated using immunochemistry.
